# Orbital Rosai–Dorfman Disease in a fifty-eight years old woman

**DOI:** 10.12669/pjms.294.3554

**Published:** 2013

**Authors:** Hui-Yan Li, Hong-Guang Cui, Xue-Yong Zheng, Guo-Ping Ren, Yang-Shun Gu

**Affiliations:** 1 Hui-Yan Li, Ophthalmology, The First Affiliated Hospital, School of Medicine, Zhejiang University, Hangzhou, China.; 2 Hong-Guang Cui, Ophthalmology, The First Affiliated Hospital, School of Medicine, Zhejiang University, Hangzhou, China.; 3 Xue-Yong Zheng, General Surgery, The Affiliated Sir Run Run Shao Hospital, School of Medicine, Zhejiang University, Hangzhou, China.; 4 Guo-Ping Ren, Pathology, The First Affiliated Hospital, School of Medicine, Zhejiang University, Hangzhou, China.; 5 Yang-Shun Gu, Ophthalmology, The First Affiliated Hospital, School of Medicine, Zhejiang University, Hangzhou, China.

**Keywords:** Orbital Inflammation, Rosai–Dorfman disease

## Abstract

Rosai–Dorfman disease (RDD) is rare and characterized by histiocytic proliferation and massive cervical lymphadenopathy. About 40% of patients have extra-nodal involvement. Opthalmic involvement is seen in 10% of cases. A case of orbital Rosai Dorfman disease in a 58 years old woman is presented here, who was misdiagnosed as orbital inflammatory disease initially. The patient did not respond to a course of oral prednisolone. Then complete surgical excision of the mass was performed and the histopathological examination was consistent with a diagnosis of RDD.

## INTRODUCTION

Rosai-Dorfman disease is a non-neoplastic disease of unknown etiology observed mainly in children and adolescents, affecting mainly the lymph nodes.^1^ About 40% of patients have extranodal manifestation in the upper respiratory tract, salivary gland, skin, bone, meninges and central nervous system and testis.^2^^-^^4^ Orbital involvement in the absence of lymphadenopathy is relatively uncommon. The diagnosis depends on the histopathology and immunohistochemitry. We report a case of orbital Rosai Dorfman disease in a 58 years old woman, who was misdiagnosed as orbital inflammatory disease initially.

## CASE REPORT

**Fig.1:**
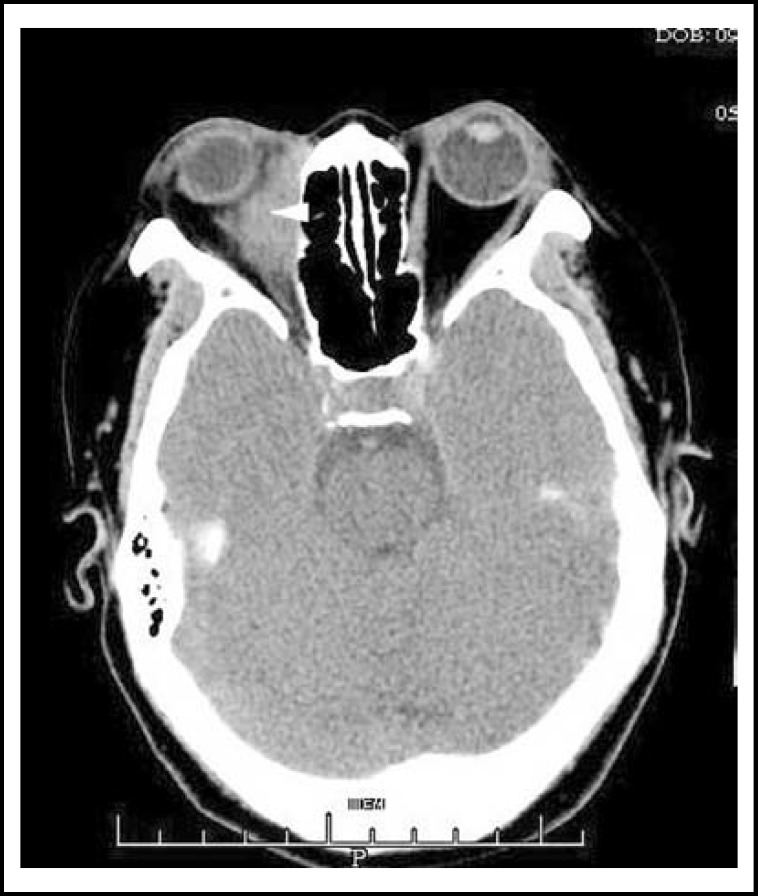
CT image showing a homogenous mass in right orbit (Arrow

The patient was a 58-year old woman, who complained of exophthalmos for 30 years and developed a swollen mass of right lower-eyelid for two years. Her visual acuity was 20/20 in both eyes. Ocular pressure was 20mmHg in right eye and 22mmHg in left eye. The measurement of exophthalmos was 18mm in right eye and 14mm in left eye. Examination revealed a large, tough, non-tender mass in right lower eyelid measured 2.0cm by 1.5cm by 0.8cm and that in upper eyelid measured 1.0cm by 0.5cm by 0.5cm. Anterior segment examination, fundoscopy and systemic examination were essentially normal. A computerized tomography (CT) scan of the orbits done at that time diagnosed inflammatory pseudotumor, shown a homogenous mass in right orbit (Fig.1).

**Fig.2:**
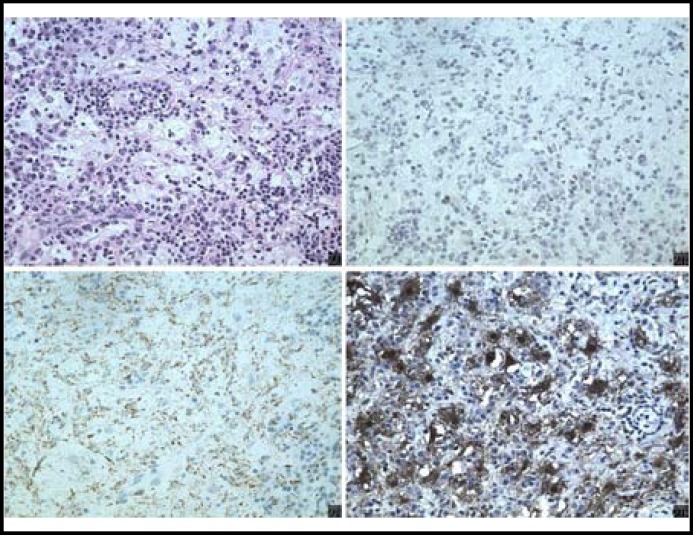
Pathological section of the patient demonstrating emperipolesis (A) (arrow), Immunohistochemically, the large histiocytes reacted negative to CD1a (B) and positive to CD68 (C) and S-100 protein (D).

A preoperative diagnosis of inflammatory pseudotumor was made. But she gave no response to a course of oral prednisolone. Then complete surgical excision of the mass was performed under general anaesthesia. Histopathological examination revealed infiltrates composed of histiocytes, lymphocytes and plasma cells. Histiocytes with indecisive outlines were large. A characteristic finding was the presence of lymphocytes engulfed within the histiocytic cytoplasm, a feature called emperipolesis. Immunohistochemically, the large histiocytes reacted strongly and positively to S-100 protein and CD68, but negative to CD1a (Fig.2). So the histopathological examination was consistent with a diagnosis of RDD. Recurrence didn’t occur by two years follow-up.

This study was approved by the institutional ethics committee of The First Affiliated Hospital, School of Medicine, Zhejiang University. Informed consent was obtained from the patient and her family.

## DISCUSSION

Rosai-Dorfman disease is a rare and non-hereditary histiocytic proliferative disorder, recognized by Rosai and Dorfman. They described four cases called “sinus histiocytosis with massive lymphadenopathy” initially. It usually presents with massive painless bilateral cervical lymphadenopathy. Opthalmic involvement is seen in 10% of cases.^5^ Typically, it involves the intraconal space in the orbit and runs a slow infiltrative and cicatricial course.^6^ The exact pathogenesis of RDD is unknown. However, some studies suggest that human herpes virus type 6 (HHV-6) and Epstein-Barr virus may play a role.^7^

The differential diagnosis of RDD includes Langerhans’cell histiocytosis, inflammatory pseudotumor and lymphoma.^8^^,^^9^ RDD is relatively benign and may take years to develop. Only tumors affecting organ functions are considered for treatment. The diagnosis of RDD depends on the histopathological and immunohistochemical confirmation. The tissue consist of numerous histiocytes, lymphocytes, plasma cells and neutrophile granulocytes. The polygon or elliptic histiocytes are rich of cytoplasma, which usually swallow some whole lymphocytes, plasma cells and neutrophile granulocytes. Furthermore, the tissue shows immunoreactivity for CD68 and S100, but negative for CD1a which is often present in Langerhans’cell histiocytosis.

Our patient was an elderly woman, and preoperative CT scan presented an inflammatory tumor, the preoperative diagnosis was not definite. A definite diagnosis of RDD requires histopathological and immunohistochemical confirmation. Therefore when patient is suspected of idiopathic orbital inflammation,a biopsy is considerd. Bijlsma demonstrated biopsy to be safe in not delaying diagnosis of malignancies and efficient in providing a rapid diagnosis.^10^ As for therapeutic options, such as surgical excision, radiation therapy, corticosteroid and other immunosuppressive therapies should be considered.^11^ When the patients are treated with corticosteroids, long-term follow-up is required because of the potentially vision-threatening complications and the probable recurrence. Our patient did not respond to corticosteroids and surgical excision was curative without recurrence by two years follow-up. Hence at present the surgical resection is the first choice for treatment of orbital rosai-dorfman disease.
